# The Purkinje–myocardial junction is the anatomic origin of ventricular arrhythmia in CPVT

**DOI:** 10.1172/jci.insight.151893

**Published:** 2022-02-08

**Authors:** Daniel J. Blackwell, Michela Faggioni, Matthew J. Wleklinski, Nieves Gomez-Hurtado, Raghav Venkataraman, Chelsea E. Gibbs, Franz J. Baudenbacher, Shiaoching Gong, Glenn I. Fishman, Patrick M. Boyle, Karl Pfeifer, Bjorn C. Knollmann

**Affiliations:** 1 Vanderbilt Center for Arrhythmia Research and Therapeutics, Division of Clinical Pharmacology, Department of Medicine, Vanderbilt University School of Medicine, Nashville, Tennessee, USA.; 2 Department of Pharmacology and; 3 Department of Biomedical Engineering, Vanderbilt University, Nashville, Tennessee, USA.; 4 Department of Bioengineering, University of Washington, Seattle, Washington, USA.; 5 Laboratory of Molecular Biology, Rockefeller University, New York, New York, USA.; 6 Leon H. Charney Division of Cardiology, Department of Medicine, New York University School of Medicine, New York, New York, USA.; 7 Institute for Stem Cell and Regenerative Medicine and; 8 Center for Cardiovascular Biology, University of Washington, Seattle, Washington, USA.; 9 Division of Intramural Research, Eunice Kennedy Shriver National Institute of Child Health and Human Development, NIH, Bethesda, Maryland, USA.

**Keywords:** Cardiology, Arrhythmias, Calcium signaling, Genetic diseases

## Abstract

Catecholaminergic polymorphic ventricular tachycardia (CPVT) is an arrhythmia syndrome caused by gene mutations that render RYR2 Ca release channels hyperactive, provoking spontaneous Ca release and delayed afterdepolarizations (DADs). What remains unknown is the cellular source of ventricular arrhythmia triggered by DADs: Purkinje cells in the conduction system or ventricular cardiomyocytes in the working myocardium. To answer this question, we used a genetic approach in mice to knock out cardiac calsequestrin either in Purkinje cells or in ventricular cardiomyocytes. Total loss of calsequestrin in the heart causes a severe CPVT phenotype in mice and humans. We found that loss of calsequestrin only in ventricular myocytes produced a full-blown CPVT phenotype, whereas mice with loss of calsequestrin only in Purkinje cells were comparable to WT mice. Subendocardial chemical ablation or restoration of calsequestrin expression in subendocardial cardiomyocytes neighboring Purkinje cells was sufficient to protect against catecholamine-induced arrhythmias. In silico modeling demonstrated that DADs in ventricular myocardium can trigger full action potentials in the Purkinje fiber, but not vice versa. Hence, ectopic beats in CPVT are likely generated at the Purkinje–myocardial junction via a heretofore unrecognized tissue mechanism, whereby DADs in the ventricular myocardium trigger full action potentials in adjacent Purkinje cells.

## Graphical abstract

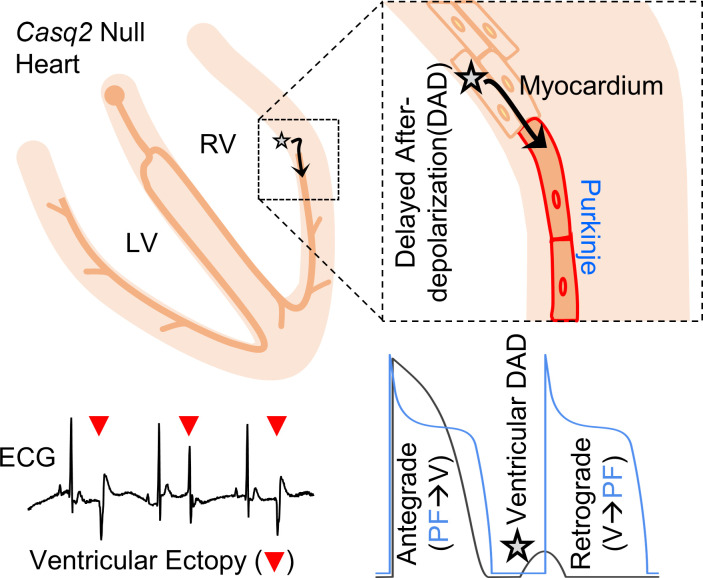

## Introduction

Catecholaminergic polymorphic ventricular tachycardia (CPVT) is an inherited disorder characterized by emotional- or physical stress-induced arrhythmias in structurally normal hearts (1). The more common autosomal-dominant form has been linked to mutations in the gene encoding the cardiac Ca release channel (*RYR2*) (2, 3). A less common but more severe autosomal-recessive form is caused by loss-of-function mutations in the gene encoding cardiac calsequestrin (*CASQ2*), the major Ca-binding protein in the sarcoplasmic reticulum (SR) (4, 5). Both *RYR2* and *CASQ2* mutations cause spontaneous premature SR Ca releases in ventricular myocytes (6, 7) that facilitate the generation of delayed afterdepolarizations (DADs) and focal ventricular arrhythmias (5, 8). Hyperactive RYR2 channels and an increased rate of spontaneous Ca release have also been observed in ventricular myocytes isolated from failing human hearts (9, 10) and animal models of heart failure (11). Because increased RYR2 activity and spontaneous diastolic Ca release is an accepted cellular mechanism for the generation of ectopic ventricular beats, CPVT can be considered a model to study Ca-triggered ventricular arrhythmia (12).

On the basis of results of experimental and modeling studies, researchers have suggested that Purkinje cells in the cardiac conduction system are the cellular source responsible for arrhythmia generation in CPVT (13–15). Purkinje cells have differential protein expression (e.g., ion channels) and structural properties (lack of transverse tubules) that make them particularly prone to develop spontaneous Ca releases (16, 17). Modeling studies have demonstrated increased sodium load and susceptibility to Ca overload, enhanced SR load, and a lower action potential (AP) threshold. Experiments in isolated Purkinje cells and intact hearts from the *Ryr2/Ryr2^R4496C^* mouse model have suggested that the Purkinje cells in the cardiac conduction system are the cellular source responsible for triggering CPVT (14, 15, 17). On the other hand, ventricular cardiomyocytes carrying CPVT mutations also exhibit spontaneous Ca release in response to catecholamine challenge and can generate DADs and spontaneous APs (6, 7). Optical mapping, commonly used to study the anatomic origin of ventricular arrhythmias, cannot resolve the Purkinje cells from the surrounding ventricular cardiomyocytes in the subendocardium. Thus, the anatomic and cellular origins of focal activation in CPVT remain uncertain.

To answer this question experimentally, we used an established *Casq2^–/–^* mouse model of CPVT (7). *Casq2* null mutations cause a severe CPVT phenotype in mice and humans. We aimed to achieve the following objectives: (a) determine the cellular origin of ventricular ectopy, using a tissue-targeted genetic approach that selectively ablates *Casq2* gene expression either in the specialized conduction system or in the ventricular working myocardium; (b) determine the anatomic origin of ventricular ectopy in the *Casq2^–/–^* mouse CPVT model, using optical voltage mapping of isolated hearts; and (c) model the Purkinje–myocardial junction and determine if and how subthreshold DADs generate ectopic beats.

The results of our experiments indicate that polymorphic ventricular tachycardia (VT) due to loss of Casq2 is triggered by cardiomyocytes located in subendocardial working myocardium and not by Purkinje cells in the specialized conduction system, as previously suggested. Our modeling data suggest that the ectopic beats in CPVT are generated at the Purkinje–myocardial junction via a hitherto unrecognized tissue mechanism, whereby subthreshold membrane depolarizations in the ventricular myocardium (VM) trigger full APs in the adjacent Purkinje fiber (PF).

## Results

### Generation of Purkinje cell–specific and VM-specific Casq2^–/–^ mouse models.

**Figure 1:**
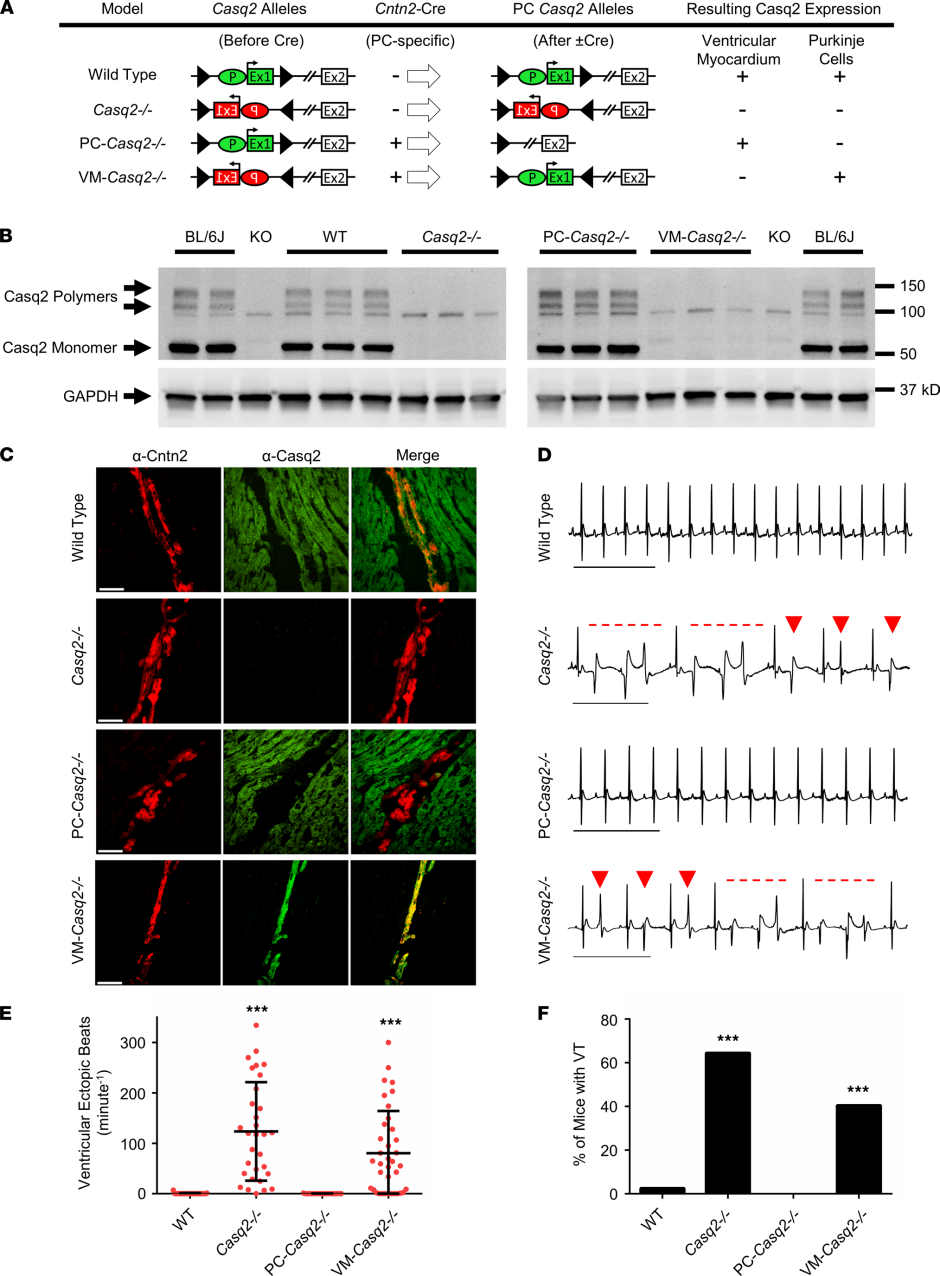
Generation and characterization of cardiac Casq2 tissue-specific mouse models. (**A**) Each mouse model is shown with its representative *Casq2* alleles before and after Cre expression in the Purkinje cells (PCs) and resulting Casq2 protein expression in the VM and PCs. The *Casq2* floxed allele contains the promoter (P) and exon 1 (Ex1) in either the forward or reverse gene orientation flanked by loxP sites (triangles). Cre expression (if present) flips the orientation of the promoter and exon 1, resulting in 4 models: WT (global Casq2^+/+^ expression); *Casq2^–/–^* (global *Casq2^–/–^*); PC-*Casq2^–/–^* (Casq2 knocked out only in PCs); and VM-*Casq2^–/–^* (Casq2 knocked out only in the VM). *Cntn2* is only expressed in PCs. (**B**) Western blots from Casq2 tissue-specific mouse hearts. C57BL/6J (BL/6J) was included as a positive control. Casq2 null (KO) is an independent Casq2 germline deletion model (7) and was included as a negative control (the same Bl/6J and KO samples were loaded on both gels). GAPDH was used as a loading control. (**C**) Representative immunostaining for Cntn2 and Casq2 from sectioned mouse hearts. Scale bar: 50 μm. (**D**) Representative ECG traces from each mouse model after i.p. administration of 3 mg/kg ISO plus 60 mg/kg caffeine. Arrows denote premature ventricular contractions, and dashed lines denote episodes of VT. Scale bar: 500 ms. Quantification of VEBs (**E**) and VT (**F**) incidence in 8–38-week-old mice. WT sample, *n* = 39; Casq2KO, *n* = 31; PC-*Casq2^–/–^*, *n* = 16; VM-*Casq2^–/–^,* *n* = 28. (**E**) Data reported with mean ± SD. ***P < 0.001 versus WT or PC-*Casq2^–/–^* by Kruskal-Wallis test followed by Dunn’s multiple comparisons post hoc test (**E**) or the Fisher exact test (**F**).

To selectively delete Casq2 expression either in Purkinje cells (PC-*Casq2^–/–^* mice) or in the VM (VM-*Casq2^–/–^* mice), mice with conditional deletion or with conditional rescue *Casq2* alleles (18) were crossed with mice expressing Cre recombinase under control of the contactin-2 (*Cntn2*) promoter (see Methods section). Immunostaining and Western blotting from selected hearts confirmed successful Purkinje cell– or cardiomyocyte-specific Casq2 deletion in our tissue-targeted murine models (Figure 1, B and C, and Supplemental Figure 1; supplemental material available online with this article; https://doi.org/10.1172/jci.insight.151893DS1).

### Arrhythmia susceptibility in tissue-targeted Casq2^–/–^ mice.

Arrhythmia susceptibility was tested in anesthetized, 8–38-week-old mice (Table 1) injected i.p. with 3 mg/kg isoproterenol (ISO) or 3 mg/kg ISO plus 60 mg/kg caffeine during continuous surface ECG recording. As expected, WT mice had no ventricular arrhythmias (Figure 1, D–F). Global KO of Casq2 caused a full-blown CPVT phenotype characterized by premature ventricular contractions (PVCs) and bidirectional and polymorphic VT (Figure 1, D–F), comparable to what we have previously observed in a germline whole-heart *Casq2^–/–^* model (7). One Casq2 KO mouse had no PVCs (Figure 1E) but had supraventricular tachycardia immediately after catecholamine injection, an observation we have noted may protect these mice from ventricular ectopy (19, 20).

### Deletion of Casq2 in the VM is both necessary and sufficient to cause a CPVT phenotype; deletion of Casq2 in the His-Purkinje system is not.

To determine whether loss of Casq2 only in Purkinje cells is sufficient to induce a CPVT phenotype, we tested arrhythmia susceptibility in our selective *Casq2^–/–^* models. When Casq2 was knocked out only in the Purkinje cells (PC-*Casq2^–/–^*), no ventricular arrhythmias were observed despite injection with both 3.0 mg/kg ISO and 60 mg/kg caffeine (Figure 1, D–F). Normally, 1.5 mg/kg ISO alone is sufficient to induce a CPVT phenotype in global *Casq2^–/–^* mice (7). These results suggest that Casq2 loss in the conduction system alone is not sufficient to cause ventricular arrhythmias.

To test whether the ventricular cardiomyocytes are the cellular origin for CPVT, we examined arrhythmias in mice in which Casq2 was selectively knocked out in the VM but maintained in the Purkinje cells. VM-*Casq2^–/–^* mice had significant ventricular arrhythmias, with similar arrhythmia burden as whole-heart *Casq2^–/–^* mice (*P* = 0.29; Figure 1, E and F). Taken together, these data indicate that loss of Casq2 in the ventricular working myocardium is both necessary and sufficient to cause ventricular ectopy in CPVT, even when Casq2 is appropriately expressed in the conduction system.

### Optical voltage mapping indicates focal origin of ventricular arrhythmias in Casq2^–/–^ mouse hearts.

**Figure 2:**
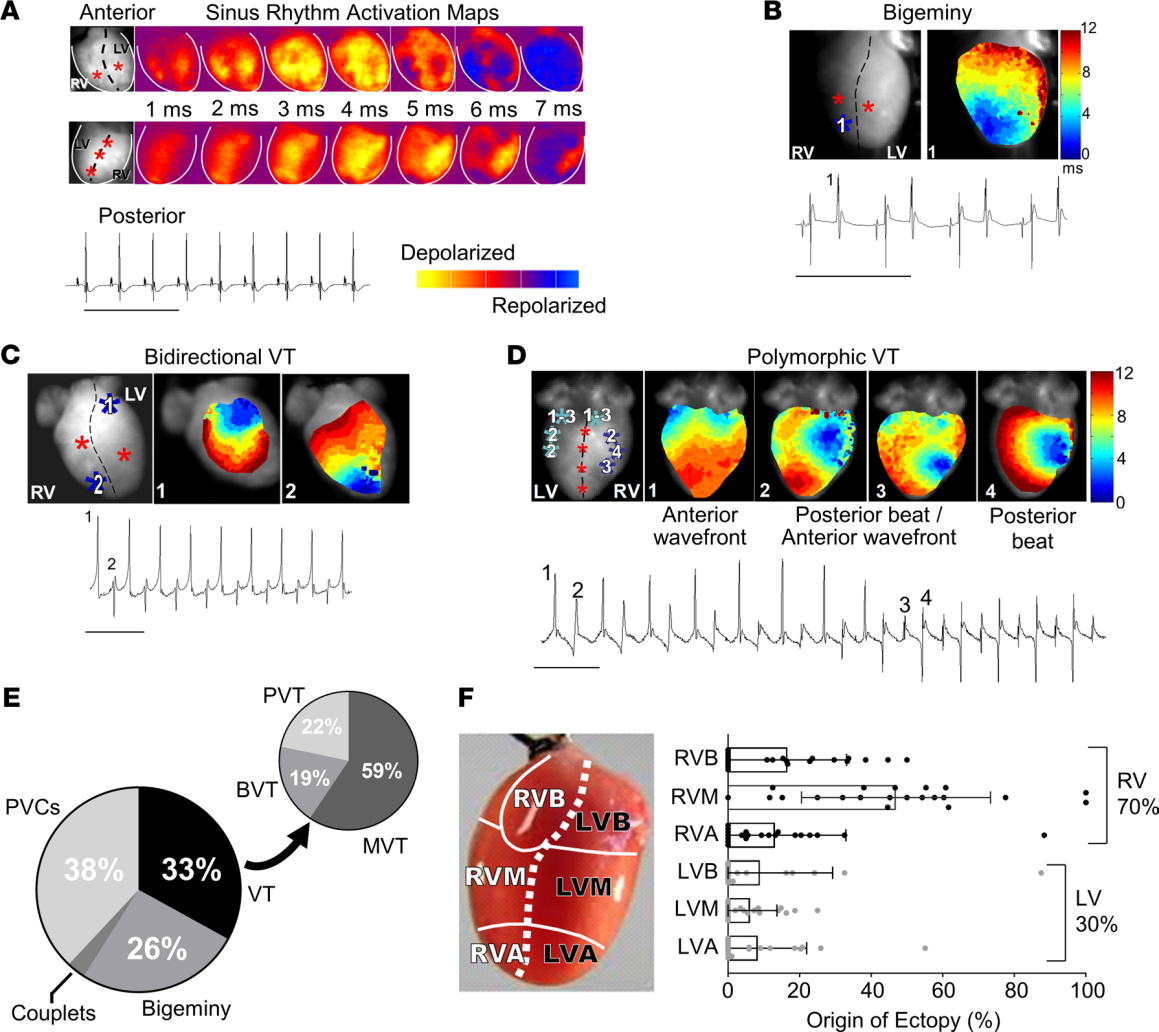
Ex vivo optical mapping and continuous ECG recording in Langendorff-perfused Casq2 null mouse hearts. (**A**) ECGs were continuously recorded while optical voltage maps were acquired from anterior and posterior epicardial surfaces during sinus rhythm. Sinus rhythm epicardial breakthroughs are denoted by red stars in the left-most panel. (**B**) Example temporal activation maps and associated ECG traces of bigeminy; (**C**) bidirectional VT; and (**D**) polymorphic VT after perfusion of a 100 nM ISO bolus. Ectopic foci are denoted by blue stars and indicated numerically on the accompanying ECG traces. (**E**) Classification of arrhythmia episodes from 8 hearts captured by ECG and optical mapping (*n* = 246 total episodes). BVT, bidirectional VT; MVT, monomorphic VT; PVC, premature ventricular contraction; PVT, polymorphic VT. ECG scale bars: 500 ms. (**F**) Quantification of the site of epicardial breakthroughs (*n* = 21 for each group) during voltage mapping from the same recordings as in **E**. Data reported as mean ± SD. R/LVB, right/left ventricular base; R/LVM, mid right/left ventricle; R/LVA, right/left ventricle apex.

To examine the anatomic origin of ventricular ectopy in CPVT, hearts were isolated from *Casq2^–/–^* mice and perfused in Langendorff mode with oxygenated Tyrode’s solution and the transmembrane potential dye di-4-aminonaphthylethenylpyridinium (ANEPPS). Voltage mapping showed normal sinus rhythm bundle branch breakthroughs at baseline (Figure 2A). Upon stimulation with perfused ISO, the hearts developed mono- and multifocal ventricular arrhythmias in the form of single PVCs, couplets, bigeminy, bidirectional VT, and polymorphic VT (Figure 2, B–D). All ventricular arrhythmias were focal in origin without any evidence for reentrant circuits. The focal activity originated from both ventricles, with right ventricle (RV) foci significantly more common than left ventricle (LV) foci (Figure 2, E and F), which mirrors observations from humans diagnosed with CPVT (21). Importantly, the breakthrough sites of all ventricular ectopic beats (VEBs) were outside and distinct from the normal sinus rhythm breakthrough sites of the left and right bundle branches. This result corroborates the results from our tissue-targeted in vivo experiments (Figure 1) and further confirms that the ventricular ectopy in CPVT does not originate exclusively from Purkinje cells in the bundle branches, as previous modeling studies had suggested (14, 22).

### Endocardial ablation prevents ventricular arrhythmia in Casq2^–/–^ mouse hearts.

**Figure 3:**
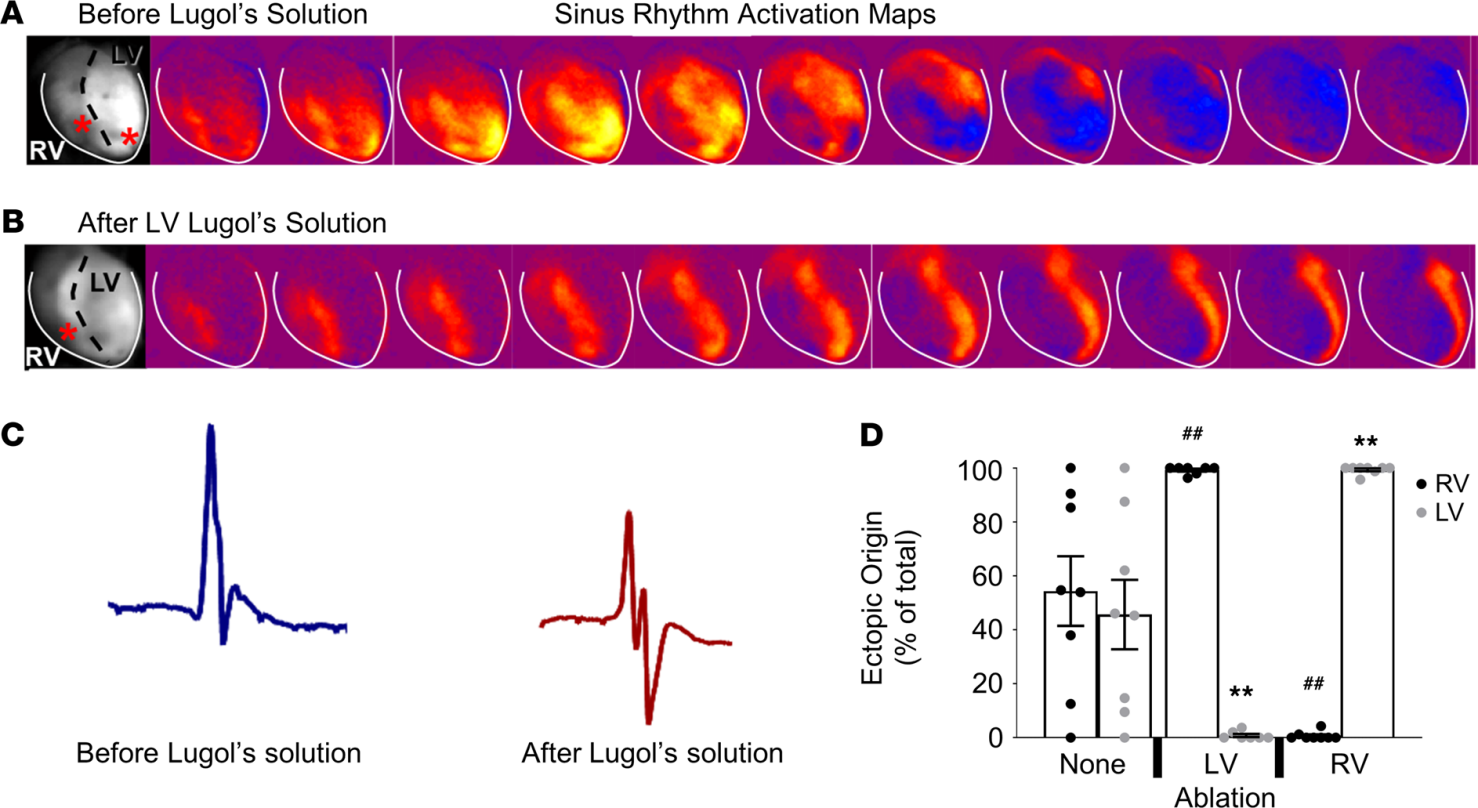
Endocardial ablation reduces arrhythmia burden in Casq2 null hearts. Lugol’s solution was injected either into the LV or RV to ablate the endocardial wall. Optical mapping before (**A**) and after (**B**) injection of Lugol’s solution into the LV. Breakthroughs are denoted in the left-most panels by red stars. Activation of the LV epicardium was maintained even after endocardial ablation. (**C**) Representative QRS waveform morphology before and after injection with Lugol’s solution. (**D**) Origin of VEBs after injection of vehicle or Lugol’s solution into the LV or RV. Arrhythmias were stimulated by perfusion with 100 nM ISO (*n* = 8 hearts/group). Data are reported as mean ± SD. ^##^*P* < 0.01 by 2-sided Student’s *t* test when comparing ectopic origin percentage from the RV with that of no ablation; **P < 0.01 by 2-sided Student’s *t* test when comparing ectopic origin percentage from the LV with that of no ablation.

To identify the origin of ventricular ectopy within the working VM, we first mapped ventricular activation of the epicardial surface of *Casq2^–/–^* hearts during sinus rhythm and during ISO-induced ventricular arrhythmias. Then, we injected Lugol’s solution into the LV or RV to chemically ablate the endocardial surface and underlying myocardium and repeated the ISO challenge (Figure 3, A and B). All *Casq2^–/–^* hearts displayed loss of the left-sided breakthrough during sinus activation after LV endocardial ablation, thereby establishing the successful disruption of the conduction system and endocardial activation in the LV. However, the activation wavefront originating from the RV breakthrough was still homogeneously conducted on the epicardial surface of both ventricles (Figure 3B). The QRS complex duration after the procedure was significantly prolonged (10.5 ± 0.4 ms before LV ablation vs. 13.7 ± 0.9 ms after LV ablation), with a QRS morphology consistent with a left bundle branch block (Figure 3C). Importantly, the endocardial lesion produced by the chemical ablation did not increase the incidence of ventricular ectopy in 8 WT hearts, ruling out chemical ablation as a cause of arrhythmogenesis. After LV endocardial ablation in *Casq2^–/–^* hearts, VEBs originating from the LV were almost completely suppressed (only 0.4% of the total number of VEBs in Lugol’s solution–treated hearts) and RV VEBs accounted for 99.6% of all ectopic beats recorded (Figure 3D). Conversely, when the RV endocardium was ablated, the RV breakthrough for sinus beats was abolished and all ectopic ventricular activity was generated from the LV (Figure 3D). Although chemical ablation by Lugol’s solution does not discriminate between the ventricular conduction system and the working endocardium, these results, together with the evidence from the selective Casq2 KO models (Figure 1), indicate that the subendocardial myocytes are the likely cellular source of ventricular ectopy.

### Juxta-Purkinje ventricular cardiomyocytes located in the subendocardium are the cellular origin for CPVT.

**Figure 4:**
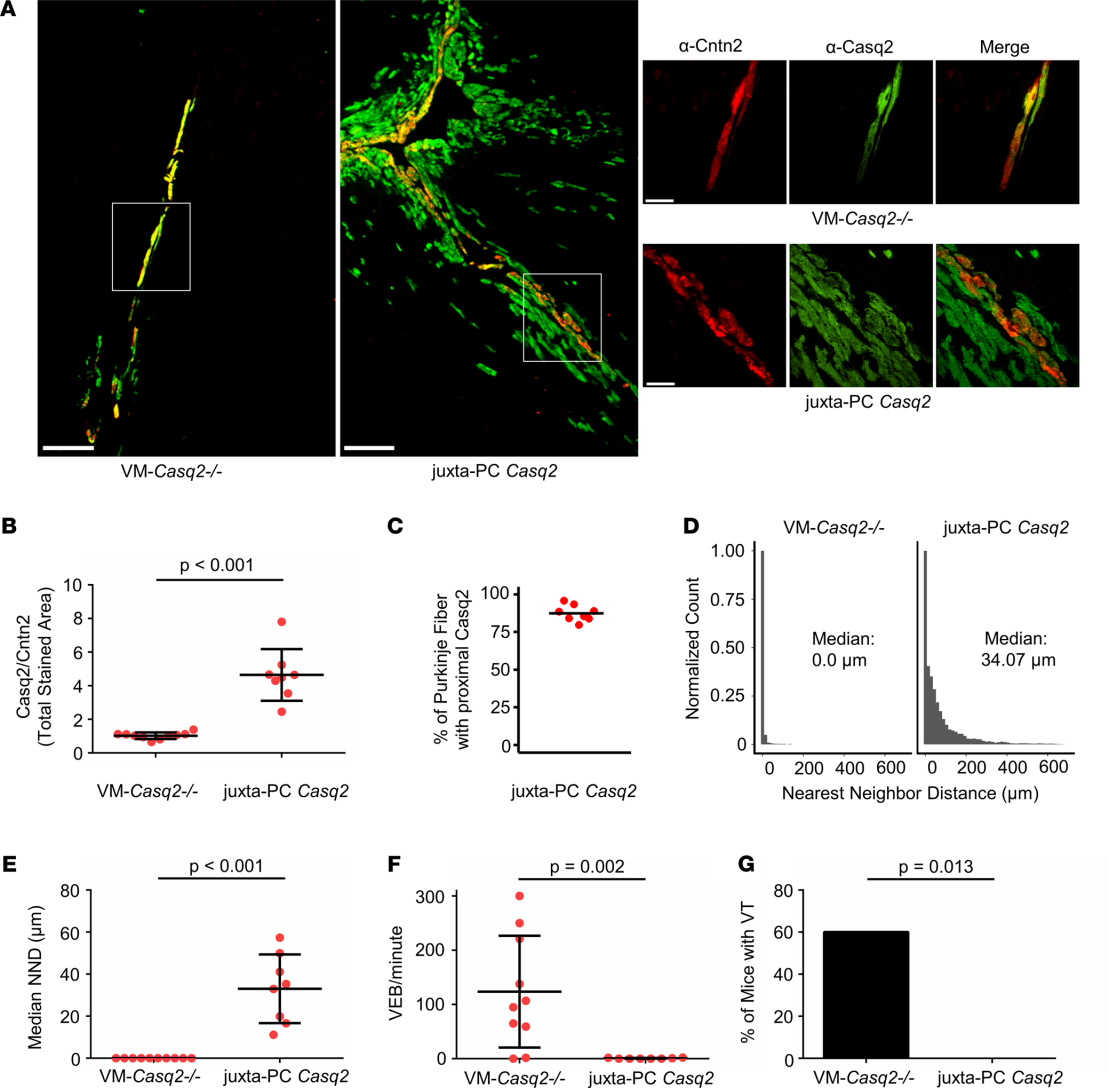
Casq2 expression in subendocardial ventricular myocytes juxtaposed to Purkinje cells reduces PVC burden and prevents arrhythmia. (**A**) Immunostaining for Cntn2 (a Purkinje cell marker) and Casq2 in selected hearts from VM-*Casq2^–/–^* mice. Scale bar: 200 μm. A subset of mice expressed Casq2, in addition to the Purkinje cells, also in ventricular myocytes next to Purkinje cells, denoted as “juxta-PC Casq2” (see top right image in **A**). Other mice co-expressed Casq2 only in Cntn2-positive cells (see lower right-side image in **A**). Scale bar in right-side images: 50 μm. (**B**) Ratio of Casq2 to Cntn2-positive immunostaining in hearts categorized as VM-*Casq2^–/–^* or juxta-PC Casq2 by a reviewer blinded to the genotype. (**C**) Percentage of Cntn2-labeled fibers having contiguous Casq2 staining in ventricular myocytes juxtaposed to the fiber. (**D**) NND distributions for Casq2-positive immunostaining relative to Cntn2-positive immunostaining. Data are displayed in 15 μm bins (individual distributions are shown in Supplemental Figure 2). (**E**) Median NND for each heart. (**F**) VEB and (**G**) VT incidence (>2 consecutive VEBs); *n* = 10 and 8 hearts/group, respectively. (**B**, **D**, and **E**) Data are reported with mean ± SD and compared using a 2-sided Mann-Whitney test. (**F**) Data were compared using the Fisher exact test.

During our investigation, we noted that several VM-*Casq2^–/–^* mice had no ventricular ectopy in vivo (Figure 1, E and F). To determine whether Casq2 was completely knocked out in the ventricular myocytes of these mice, hearts were examined by co-immunostaining for Casq2 and Cntn2 to identify Purkinje cells. We found that, alongside Casq2 expression in the conduction system, some VM-*Casq2^–/–^* hearts still expressed Casq2 in ventricular myocytes juxtaposed to the conduction system (Figure 4A), an expression pattern we termed “juxta-Purkinje” Casq2. A reviewer blinded to the genotype classified hearts on the basis of the Casq2 immunostaining relative to Cntn2 staining as either complete VM KO or juxta-Purkinje Casq2 (Figure 4, A and B). Hearts classified as having juxta-Purkinje Casq2 expression had Casq2-positive myocytes almost contiguous with the PFs (mean, 87.4%; Figure 4C). To quantify the relative colocalization in each group, we calculated the nearest neighbor distance (NND) for each positive Casq2 pixel relative to the nearest Cntn2 pixel. The median NND in complete VM KO hearts was 0.0 μm (perfect colocalization), whereas it was 34.07 μm in hearts classified as having juxta-Purkinje Casq2 expression (Figure 4, D and E; individual NND distributions for each heart are shown in Supplemental Figure 2). As shown by the NND distributions, ventricular cardiomyocytes that expressed Casq2 were only near Purkinje cells. Incidence of positive staining for Casq2 decreased as distance from the nearest Purkinje cell increased, showing that additional Casq2 expression was only in the subendocardial myocytes juxtaposed to the Purkinje cells. No Casq2 expression was observed in the epicardial tissue of any heart.

Notably, mice expressing Casq2 in myocytes juxtaposed to Purkinje cells had significantly lower rates of ectopy (*P* = 0.006) and accounted for most of the zero values within the VM-*Casq2^–/–^* (Figure 1E) that were immunostained (Figure 4, F and G). The arrhythmia rates in the complete VM KO group were essentially the same as for the whole-heart *Casq2^–/–^* model (*P* = 0.94), whereas the juxta-Purkinje group had significantly fewer ectopic beats and reduced arrhythmia burden compared with the whole-heart *Casq2^–/–^* group (*P* < 0.001). Although it is unclear why, in a subset of VM-*Casq2^–/–^* mice, Cre was turned on in cardiomyocytes adjacent to Purkinje cells, this serendipitous finding provides direct evidence that subendocardial ventricular myocytes near PFs are the cellular origin for CPVT. Expression of Casq2 in VM juxtaposed to Purkinje cells protected against arrhythmias, whereas Casq2 expression only in the Purkinje cells did not (Table 2).

We next examined expression of markers associated with transitional cells, a cell population intermediate between Purkinje cells and ventricular cardiomyocytes (23), to determine whether juxta-ventricular myocytes represent a unique cellular subtype. In a recent study, researchers conducted transcriptomic profiling on the cardiac conduction system of developing mouse hearts to identify genes that could characterize the various parts of the conduction system (24). Transitional cells were identified by a gene expression pattern that included lower levels of Cx40, higher levels of Cx43, and high levels of copine-5 (Cpne5). Authors of other studies, though, have suggested that Cx40 levels are increased in both Purkinje and transitional cells (25).

We first examined WT hearts to determine whether any differential expression of connexin 40 (Cx40), connexin 43 (Cx43), and Cpne5 could be established in myocardium juxtaposed to the PFs. In all examined sections from WT hearts, Cx40 was only associated with the PFs, and Cx40 staining did not extend beyond the boundary defined by Cntn2 staining (Supplemental Figure 3). Conversely, Cx43 staining was observed in all Cntn2-negative juxta-Purkinje ventricular myocytes. Cpne5 staining mirrored that observed for Cx40. These findings indicate that the VM surrounding the PFs is not part of a transitional cell population.

**Figure 5:**
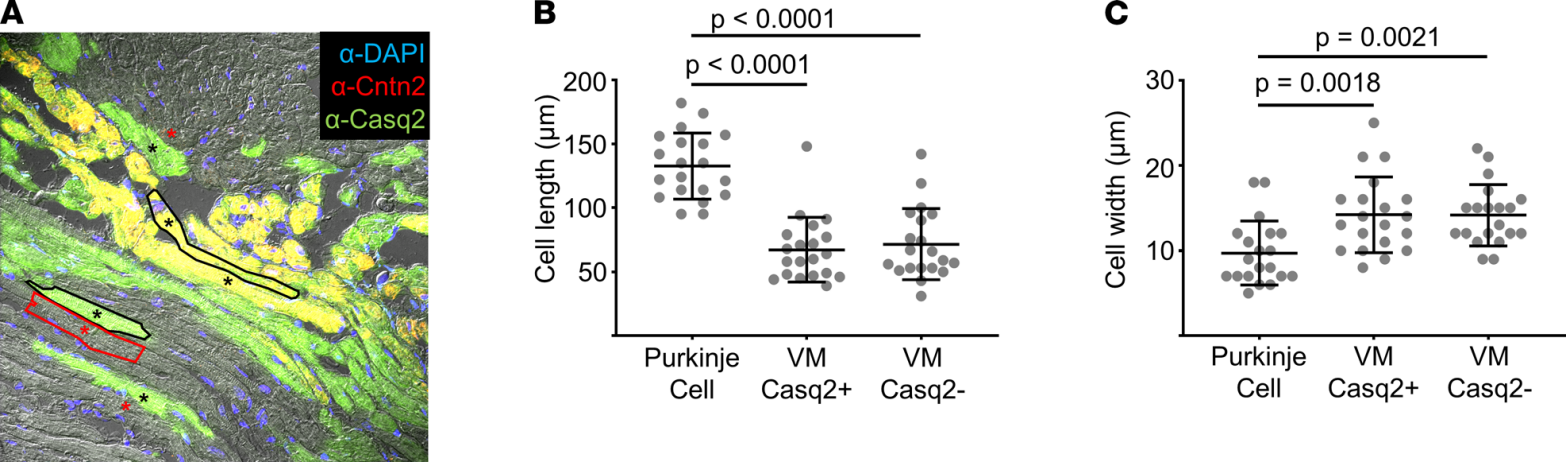
Juxta-Purkinje ventricular myocytes expressing cardiac Casq2 are morphometrically like ventricular myocytes lacking Casq2. (**A**) Representative image showing Purkinje cells stained by Cntn2 (resulting in yellow) alongside ventricular myocytes expressing Casq2 (green) or lacking Casq2 (gray). Cell boundaries are drawn for 1 cell of each type and selected cells used for analysis are marked with *. Scale bar: 50 μm. (**B**) Cell length. (**C**) Cell width. Data collected from 6 fields of view for a total of 20 cells/group. Data are reported with mean ± SD and compared using 1-way ANOVA with Tukey’s multiple comparisons test.

To determine whether cells with juxta-Purkinje Casq2 may be part of a unique transitional cell population that erroneously arises during development, we repeated the immunostaining for Cx40 and Cx43 in all 4 remaining groups (*Casq2^–/–^*, PC-*Casq2^–/–^*, VM-*Casq2^–/–^*, and juxta-Purkinje Casq2). There were no differences in Cx40 or Cx43 immunostaining, indicating that the VM expressing Casq2 is not part of the transitional cell population (Supplemental Figure 4 and Supplemental Table 1). Furthermore, morphometric analysis of juxta-Purkinje myocytes showed that they are distinct from Purkinje cells but not significantly different from adjacent ventricular myocytes (Figure 5). Hence, the juxta-Purkinje myocytes represent the VM at the Purkinje-myocardial junction.

### In silico modeling of the Purkinje–myocardial junction.

Taken together, our experimental data indicate that ectopic beats in the CPVT mouse model are generated near or at the Purkinje–myocardial junction. Given the unique geometric properties of the Purkinje–myocardial junction, which facilitates retrograde conduction (26, 27), we hypothesized that ectopic beats are triggered by subthreshold membrane depolarizations (i.e., DADs) in the juxta-Purkinje ventricular myocytes. DADs are a well-established cellular consequence of spontaneous Ca release due to activation of the electrogenic Na–Ca exchanger (28). To test this hypothesis, we used a computational model to examine the effect of the unique tissue geometry at the Purkinje–myocardial junction. Simulations were set to conditions reflecting the selective expression of Casq2 in either the Purkinje cells or VM alone (i.e., DAD-like activity in only 1 of the tissue subtypes). In the first model, we chose initial conditions in which the entire block of ventricular tissue was prone to DAD-like activity, but the coupled PF structure was not. From this starting point, we allowed electrophysiological activity in the model to evolve by itself and monitored for the incidence of retrograde excitation.

**Figure 6:**
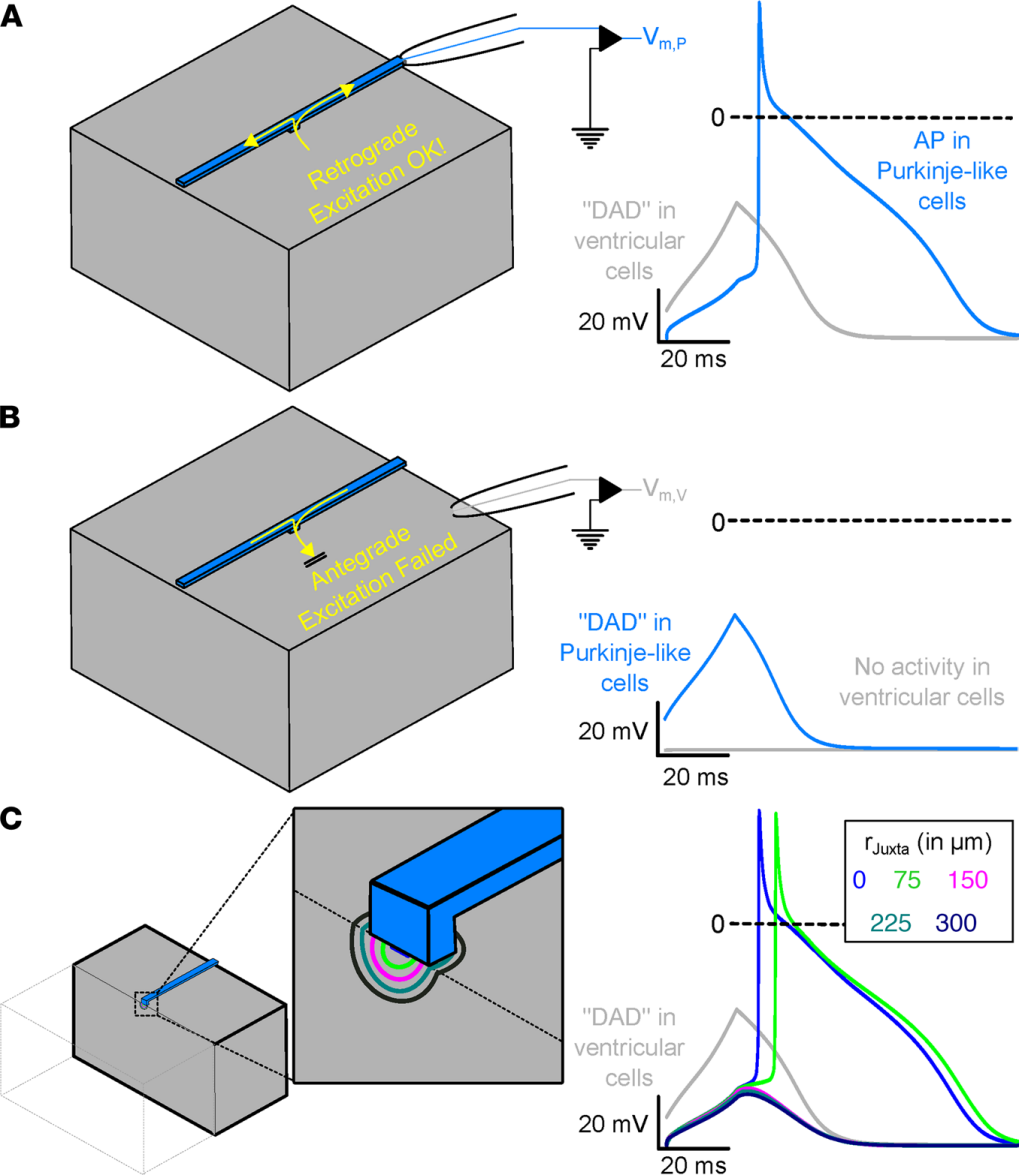
Subthreshold DAD-like activity in ventricular cells of the Purkinje–myocardial junction cause retrograde excitation of PFs. (**A**) Representation of the tissue block (left) used in the computational model. Recording electrode is illustrated for the ventricular (gray) and Purkinje (blue) tissue subtypes. The membrane voltage recording (at right) shows a ventricular DAD (gray) triggering an AP in the PF (blue). (**B**) Reciprocal experiment demonstrating that Purkinje DADs fail to generate ventricular APs. (**C**) Schematic representation of the computational model. Clipping plain and zoomed-in inset show the boundaries of the hemispherical juxta-cell region with characteristic r_Juxta_. Membrane voltage traces (right), showing DAD-like activity in ventricular tissue (gray; identical regardless of r_Juxta_ value) and the response in a coupled PF for various r_Juxta_ values. Evolution of membrane voltage over time in this model for all r_Juxta_ values can be found in Supplemental Video 1.

Retrograde excitation occurred as subthreshold DADs in the VM-triggered APs in the PFs (Figure 6A). When electrophysiological properties of the 2 regions were reversed (i.e., the conditions for DAD-like activity were in the PFs instead of the surrounding myocardium), antegrade excitation did not occur (Figure 6B). Thus, in this model, the asymmetric propensity for DAD-induced ectopic beats initiated by retrograde versus antegrade conduction is a consequence of Purkinje–myocardial junction geometry. These findings establish a mechanism for triggered activity and explain how selective KO of Casq2 only in the myocardium still leaves the PFs vulnerable to triggered activity by the DAD-prone ventricular myocyte.

To test our hypothesis that the presence of non–DAD-prone juxta-Purkinje ventricular cells could inhibit retrograde excitation, we simulated a hemispherical region around the Purkinje–ventricular junction (with radius r_Juxta_ ranging from 0 to 300 μm in steps of 75 μm; Figure 6C, left inset) of ventricular cells that did not undergo DADs. When most of the ventricular tissue (*r_Juxta_* = 0 or 75 μm) underwent a DAD-like excitation, retrograde excitation of the PF was observed, even as the local bulk of the ventricular tissue remained at sub-threshold levels. For larger values of *r_Juxta_*, the PF was buffered from DAD-like activity and retrograde excitation did not occur (Figure 6C). These observations demonstrate how expression of Casq2 within close proximity of the PFs is sufficient to prevent any DAD-like activity in the mid- or epimyocardium from retrograde excitation and illustrate that only DADs from subendocardial myocytes at the Purkinje–myocardial junction trigger APs.

## Discussion

Our experimental work supports 3 important conclusions regarding the cellular and anatomic origins of polymorphic VT in this model of CPVT. First, the cell type responsible for triggering arrhythmogenesis in CPVT is the ventricular cardiomyocyte. We found that VM-*Casq2^–/–^* mice have an arrhythmia burden equivalent to that of global *Casq2^–/–^* mice (Figure 1), whereas expression of Casq2 only in the Purkinje cells did not protect against catecholamine-induced VT. In silico modeling suggests that, at the Purkinje–myocardial junction, subthreshold ventricular DADs cause full Purkinje APs capable of propagation. In contrast, antegrade excitation from DADs in the PF does not take place.

Second, subendocardial cardiomyocytes juxtaposed to Purkinje cells are the cellular source for arrhythmogenesis. We used histologic analysis to relate the type of cells expressing Casq2 with arrhythmia susceptibility to catecholamine challenge or exercise. Subendocardial expression of Casq2 near Purkinje cells was sufficient to prevent CPVT, further supporting the modeling data that subthreshold DADs rather than APs in the VM are the underlying mechanism. Moreover, ablation of the endocardial surface confirmed previous reports that the endocardial wall, and not the epicardium, is the arrhythmogenic focus in CPVT. Finally, in silico modeling also supported the experimental findings, showing that only DADs near the Purkinje–myocardial junction trigger APs in the PF.

Third, CPVT foci predominately arise from the right side of the heart. Voltage mapping showed right-sided epicardial breakthrough in 70% of all arrhythmogenic ventricular ectopy, analogous to observations in humans with CPVT.

Evidence of the pathophysiology of CPVT has been used to better understand arrhythmogenesis in several acquired heart conditions characterized by impaired Ca trafficking, such as heart failure and hypertrophic cardiomyopathy. For this reason, the cellular origin of focal arrhythmias in CPVT has been a matter of interest and debate for years. Experimental evidence has led some investigators to conclude that Purkinje cells are the cellular foci in CPVT, based on the morphology of its trademark arrhythmia: bidirectional VT. The 180° QRS axis shift that characterizes the bidirectional pattern on the ECG suggested that the ectopic activity originated alternatively from right and left bundle branches (14). Indeed, voltage activation maps in *Casq2^–/–^* isolated hearts often show alternating right and left ventricular activation during VT. In vitro studies, highlighting the higher rates of spontaneous Ca releases in isolated Purkinje cells compared with VM, seemed to support this hypothesis (15).

However, several reports challenge this model: Observations from human patients frequently show anatomic foci outside of PFs (29) that could be successfully silenced via ablation (30). Interestingly, an inverse correlation between sudden death and arrhythmogenic focal distance from the conduction system was found in 1 study, although both the sudden death cohort and surviving cohort had foci near the conduction system (21). Additional support comes from cellular studies: Purkinje cells have a prolonged refractory period that would make them less likely to generate triggered ectopic activity in vivo during normal sinus rhythm. Our genetic approach reported here points toward the incidence of DADs in a specific subset of the working myocardium—ventricular myocytes juxtaposed to Purkinje cells—as the critical prerequisite for arrhythmia initiation. Our results from activation map experiments with ablation of the endocardial layer (Figures 2 and 3) corroborate that conclusion.

Why are subendocardial ventricular cardiomyocytes juxtaposed to Purkinje cells capable of triggering ventricular ectopy in vivo, but Purkinje cells are not? Both cell types can trigger DADs and spontaneous beats in single-cell experiments after enzymatic isolation (14, 31). Previous reports highlighted the susceptibility of Purkinje cells to Ca overload and indicated their high rate of DADs as evidence that these cells are the cellular trigger. However, as shown in our modeling experiments (Figure 6), DAD-like activity in PFs cannot excite the myocardium, whereas sub-threshold excitation of the ventricular bulk myocardium can initiate retrograde excitation of the PFs due to the favorable source-sink relationship at the Purkinje–myocardial junction. Observations of superfused preparations suggest source-sink mismatch and that the presence of a resistive barrier at Purkinje–myocardial junctions leads to longer delay times for antegrade, compared with retrograde, conduction (26, 27). Many factors at the junction influence the “safety factor” for conduction (32, 33) from PFs to ventricles and vice versa. Propagation from high- to low-conductivity tissue (i.e., PF to VM) has a high safety margin that confers a slight advantage for antegrade transmission. But this slight increase in safety margin is more than offset by the reduction of the safety factor when the electrical impulse propagates across thin-to-thick tissue expansions (i.e., at the Purkinje–myocardial junction). Thus, retrograde transmission is favored overall. The present findings suggest that these suppositions hold true in the context of subthreshold excitations (as might be caused by a DAD) able to retrogradely excite the PFs with a comfortable margin of safety, even while transmission in the opposite (antegrade) direction fails.

Although our simulation studies favor retrograde excitation by subthreshold membrane depolarizations in the VM, we cannot exclude that ectopic foci could also be caused by full ventricular AP. Any APs arising from the mid myocardium or epicardium must compete with a large sink, whereas juxta-Purkinje ventricular myocytes can more easily conduct into the nearby Purkinje cells. Regardless of whether ventricular DADs or ventricular APs are the cellular trigger, ventricular cells at the Purkinje–myocardial junction still produce an apparent activation and QRS morphology consistent with Purkinje cell origin, as previously described in activation maps. Our data showed that restoration of Casq2 at a median (nonzero) distance of 53.8 μm from the nearest Cntn2-positive (Purkinje) cells was sufficient to prevent arrhythmias, indicating that distal (epicardial) cardiomyocytes, despite lacking Casq2, are not the cellular trigger for CPVT. The observation that cardiomyocytes distal from the Purkinje–myocardial junction do not trigger ventricular ectopy (Figures 3 and 4) further supports the hypothesis that subthreshold myocardial DADs, rather than full myocardial APs, are the cellular mechanisms responsible for focal ectopic activity in CPVT.

Recent work by Flores et al. (18) suggested that concurrent loss of Casq2 in both the myocardium and Purkinje system was required to generate a CPVT phenotype. In that study, conditional deletion or rescue of Casq2 was achieved in adult mice using a tamoxifen-induced *cre* expression system under the control of an *Hcn4* promoter to target the cardiac conduction system (18). Analogous to our results, Casq2 deletion only in the cardiac conduction system was not sufficient to generate a CPVT phenotype. In contrast to the data presented here, turning on *Casq2* gene expression with *Hcn4*-*cre* in adult mice prevented catecholamine-induced VT (18). One explanation for this discrepancy could be that *Casq2* expression was also activated in the VM outside the conduction system by the *Hcn4*-*cre*. This is a distinct possibility, because although *HCN4* gene expression is much higher in the conduction system, the *HCN4* gene is expressed in the VM, especially in failing hearts (34). As shown here in Figure 4, even a small number of myocytes expressing Casq2 in the subendocardial myocardium is sufficient to prevent CPVT. Furthermore, Flores et al. (18) reported that Casq2 rescue in the sinoatrial node caused a sinus tachycardia. The association of slow sinus heart rate with CPVT susceptibility has been shown previously by our group and others (1, 2, 7, 35, 36); a fast sinus rhythm is protective against catecholamine- or exercise-induced ventricular arrhythmia (19, 20). The low rate of ventricular arrhythmias observed by Flores et al. (18) after Casq2 rescue in the sinoatrial node could be attributed to the sinus tachycardia in this model.

The identification of subendocardial cardiomyocytes juxtaposed to PFs as the cellular driver and the Purkinje–myocardial junction as the anatomic origin of focal ventricular ectopy has important mechanistic and therapeutic implications beyond CPVT. DADs caused by spontaneous Ca release are considered the underlying cellular mechanism for ventricular arrhythmias after myocardial infarction or in the failing heart, based on studies of ventricular myocytes isolated from failing human hearts (9, 10) and animal models of heart failure (11). Our data suggest that, as in CPVT, the ectopic beats in heart failure may also be generated at the Purkinje–myocardial junction via a heretofore unrecognized tissue mechanism, whereby subthreshold membrane depolarizations caused by spontaneous RyR2 Ca release in the VM generate full APs in the adjacent PF. Hence, intervention with pharmacologic agents that target RyR2 can prevent arrhythmias not only in CPVT but also in heart failure models (37).

## Methods

### Generation of tissue-specific Casq2^–/–^ mice.

The generation of conditional Casq2 has been reported (18). Briefly, to modify Casq2 expression, mice were generated with the promoter and exon 1 of *Casq2* in the forward (*fCasq2*) or reverse (*rCasq2*) orientation flanked by loxP sites. The *rCasq2* allele is similar to the modified *Casq2* allele used to generate *Casq2*-null mice in a previous report (7). However, in the reverse orientation, there are 3 important differences. First, the promoter and first exon sequences between the *loxP* sites are inverted relative to the rest of the *Casq2* gene. Thus, no functional *Casq2-*encoding RNA can be generated, making *rCasq2* effectively a null allele. Second, the *loxP* sites are inverted relative to each other (whereas in the *fCasq2* gene, they are in the conventional tandem orientation). Consequently, Cre-mediated recombination results in inversion and not deletion of the intervening sequences. Thus, the cre enzyme acts on the *rCasq2^–^* allele to restore normal gene structure. Third, the *loxP* sites used in the *rCasq2^–^* allele each carry a single point mutation. Cre-mediated recombination between these 2 *loxP* elements generates 2 new *loxP* elements: 1 WT and 1 carrying both point mutations. These 2 *loxP* elements do not recombine with each other, thus making the Cre-mediated inversion unidirectional and permanent. The *fCasq2* allele contains loxP sites in tandem orientation, resulting in Cre-mediated deletion of the intervening sequence.

To generate the *rCasq2* allele, mouse ES cells (RI line, 129SV) were transformed with linearized plasmid, pKP700. Plasmid pKP700 included a 2.1 kb 5′ homology flank and a 2.0 kb 3′ homology flank to direct insertion of a *loxP* element at –561 bp and an inverted *loxP* element and a 2.1 kb NeoR cassette at +538 bp inside of intron 1. In addition, the entire 1.1 kb region between the *loxP* elements was inverted relative to the external flanks. Plasmid pKP700 also contained a 3.0 kb *Diphtheria toxin A* gene for negative selection. G418-resistant colonies were isolated and scored for homologous recombination by PCR amplification using 1 primer from outside the flanking sequences present in pKP700 and 1 primer from within the *NeoR* cassette. Targeted clones were injected into C57BL/6J blastocysts, and chimeric animals were crossed with C57BL/6J female mice to establish the *rCasq2-Neo* line. These male mice were crossed with Rosa26-Flp transgenic female mice (Jackson Laboratory strain 003946) to remove the *NeoR* cassette and thereby generate mice carrying the *rCasq2* allele.

To generate transgenic mice expressing *cre* under the control of the Purkinje cell–specific *Cntn2* gene, we used BAC recombineering, like the approach used in the GENSAT program to generate *Cntn2-EGFP* BAC reporter mice (38). *Cntn2-cre* BAC DNA was injected into FVB/N pronuclei. Founders were screened by crossing with a floxed dTomato reporter, and 1 line with expression that best colocalized with the *Cntn2-EGFP* reporter strain (39) was identified.

All transgenic lines were backcrossed into the C57BL/6J strain at least 10 times before they were used in our studies. Mice homozygous for *fCasq2 or rCasq2* were crossed with mice heterozygous for *Cntn2-cre* to generated tissue-specific calsequestrin KO lines, as described in Figure 1.

### Arrhythmia induction in anesthetized mice.

Arrhythmia susceptibility was tested in mice with a catecholaminergic challenge during surface ECG recording. A summary of the age, weight, and sex data is provided in Table 1. Mice were selected by an individual blinded to the genotype, anesthetized with 3% (vol/vol) isoflurane inhalation, and needle electrodes were placed subcutaneously into all 4 limbs. Isoflurane was titrated to the lowest possible setting (~1.25% vol/vol) to maintain stable sedation, and ECGs were recorded using a 16-channel PowerLab with 10 kHz sampling rate (AD Instruments). After stabilization of the baseline heart rate (1–5 minutes), mice were injected i.p. with 3 mg/kg ISO plus 60 mg/kg caffeine. ECG traces were recorded for an additional 10 minutes. Arrhythmia susceptibility was quantified as VEB per minute per mouse and as the number of mice with ventricular tachyarrhythmias (>2 consecutive VEBs) in each group. ECG analysis was conducted by 2 reviewers blinded to the genotype and then compared for precision. A third observer analyzed 2 ECG traces when the discrepancy was >0.5% of the total VEB count.

### Western blotting.

Cell lysates were prepared from hearts in homogenization buffer (50 mM TRIS, 320 mM sucrose, 1 mM DTT, 0.1% IGEPAL CA-630, pH 7.0) containing 1% protease inhibitor cocktail (Sigma P8340) and phosphatase inhibitor (Sigma P0044). Two hearts from C57BL/6J and 1 heart from a congenital *Casq2^–/–^* mouse (7) were included as controls. Samples were separated on a 4%–20% polyacrylamide gel (Bio-Rad Mini-PROTEAN), transferred to a PVDF membrane, blocked in TBS with Tween (TBST) plus 5% milk for 1 hour at room temperature and incubated overnight at 4°C with primary antibody. Blots were washed 3 times in TBST and incubated with secondary antibody for 1 hour at room temperature, washed 3 times again in TBST, and then developed with ECL reagent (GE Healthcare) and imaged using a ChemiDoc MP (Bio-Rad). For calsequestrin 2 immunolabeling, blots were incubated with 1:2000 anti-calsequestrin2 (ab108289; Abcam) and 1:5000 anti-rabbit HRP conjugate (catalog W401B; Promega). For GAPDH, blots were incubated with 1:10,000 anti-GAPDH (catalog AM4300; ThermoFisher) and 1:5000 anti-mouse HRP conjugate (catalog 31430; Invitrogen). Full uncropped and unedited blots were provided to the reviewers.

### Immunostaining.

Murine hearts were isolated, hung on a Langendorff apparatus, and perfused with Tyrode’s solution for 1 minute. Hearts were dissected in half, embedded in optimal cutting temperature compound (Fisher Scientific), and flash frozen on dry ice. A cryostat (Leica CM1950) and high-profile microtome blades (Accu-Edge 4685) were used to generate 8 μm sections on charged slides (Denville M1021). Frozen sections were fixed in 2% paraformaldehyde for 20 minutes, blocked with 5% goat serum in Dulbecco’s PBS (DPBS) plus 0.4% Triton for 1 hour at room temperature and incubated with primary antibodies overnight. Casq2 (Pierce PA1-913 or Proteintech 18422-1-AP), Contactin-2 (AF4439; R&D), and GFP (9F9.F9; AbCam) antibodies were diluted in DPBS plus Triton at 1:500, 1:20, and 1:1000, respectively. Blocking solution was used as a negative control. Secondary antibodies were diluted in DPBS plus Triton at 1:400 for Casq2 and Cntn2 and at 1:500 for GFP. Slides were incubated with Alexa Fluor 488 or 568 secondary antibody for 1 hour at room temperature. Cover slides were mounted using ProLong Gold antifade with DAPI (catalog P36941; Invitrogen) and left to dry overnight. Imaging was carried out on a Zeiss LSM 880 confocal microscope (provided by Laura Dugan, Vanderbilt University). Excitation was elicited using a 488 nm Argon laser or 561 nm diode pumped solid-state laser, and emission was collected from 493–556 nm and 568–712 nm. The images were reviewed and scored by an individual blinded to the genotype and phenotype.

Casq2- and Cntn2-immunostained images were opened in ImageJ (version 1.52i) and free polygons were hand-drawn around areas with positive staining by a reviewer blinded to the genotype and phenotype. Images were converted to binary masks and exported as text image files. NNDs for corresponding Casq2 and Cntn2 masks were calculated on a pixel-by-pixel basis in R (version 3.6.0) using the nncross() function available in the *spatstat* package (version 1.59-0). Histograms were generated with a bin width of 15 μm. The percentage of the PFs having contiguous Casq2 expression in ventricular myocytes juxtaposed to the fiber was analyzed using the freehand line tool in ImageJ to measure fiber length.

### Optical mapping and ECG recordings in Langendorff-perfused hearts.

Mice were anesthetized with 5% isofluorane inhalation. After achieving a surgical plane of anesthesia, a thoracotomy was performed and the heart harvested and retrogradely perfused in the Langendorff mode with oxygenated Tyrode’s buffer (pH 7.4; 1.5 mM Ca; 37°C). Hearts were equilibrated for 5 minutes and then stained with 10–15 μL of the transmembrane potential dye di-4-ANEPPS (0.5 mg/mL in DMSO) by slow injection of dye stock into a bubble trap directly above the cannula, followed by an additional 5 minute equilibration period. The di-4-ANEPPS was excited using a diode-pumped solid-state 532 nm laser (Coherent) focused onto the heart through 4 liquid light guides, and the fluorescence signal was collected with a 14-bit, 80 × 80 pixel, 1000 frames/s charge-coupled device camera (RedShirtImaging). Hearts were imaged from the anterior epicardial surface at baseline and during arrhythmia induction.

To facilitate induction of ventricular arrhythmia, the intrinsic sinus heart rate was reduced by adding 1 μM carbachol to the perfusate. After 5 to 10 minutes of equilibration, ventricular arrhythmias were induced with repeated 0.1 mL boli of 100 nM ISO injected into the perfusion line. Ventricular arrhythmic events typically occurred within 5 to 10 seconds after a bolus injection of ISO. Optical recordings were acquired for up to 2 minutes after injection; each recording lasted 4 seconds. ECG traces were continuously recorded and were used to determine the end of an arrhythmic burst and, hence, the end of optical data acquisition.

### LV and RV endocardial ablation in Casq2^–/–^ Langendorff-perfused hearts.

After equilibration in the 37°C bath and acquisition of basal sinus activation maps, the perfused heart was arrested in cold buffer (4°C). Lugol’s solution (~30 μL) was injected into the left ventricular cavity through an incision in the left auricle in 8 *Casq2^–/–^* and 8 WT hearts. Both the endocardial and epicardial surfaces were thoroughly washed with buffer before the heart was repositioned in the warm bath. The ablation was considered successful if the left ventricular breakthrough of sinus activation disappeared while the right-sided breakthrough was still present and conduction of the activation wavefront on the epicardial surface of both ventricles remained unchanged after the procedure. The incidence of ectopic activity in Lugol’s solution–treated *Casq2^–/–^* hearts was compared with that of *Casq2^–/–^* hearts undergoing the same procedure (i.e., equilibration, immersion in 4°C buffer, auricle incision) but receiving an injection of vehicle instead of Lugol’s solution. The same experiment was repeated on 8 other *Casq2^–/–^* mice with RV endocardial ablation with Lugol’s solution.

After the experiments with endocardial ablation, the hearts were perfused with triphenyl tetrazolium chloride dye, sectioned, and imaged. The depth of the endocardial ablation in the thickness of the LV wall was measured as the ratio of purple-stained to unstained (necrotic) tissue. Intact *Casq2^–/–^* hearts receiving an intraventricular injection of vehicle were used as controls.

### Analysis of optical data.

Optical data analysis was performed using custom algorithms implemented in MATLAB (Mathworks). Recordings were first temporally filtered with a 3-frame running-average and spatially filtered with a 3 × 3 pixel Gaussian low-pass filter. Fluorescence maps were generated by calculating the difference in fluorescence between the frame of interest and a background frame selected when the heart was fully repolarized. Isochronal activation maps were generated using an automated algorithm that scanned through user-defined time intervals to calculate the AP activation at each point; the activation time at each point was then plotted to generate the map. AP activation was defined as the time at which the absolute rate of change of fluorescence was maximal during the AP upstroke.

### Arrhythmia analysis in isolated hearts.

ECG records were reviewed and VEB identified on the basis of standard criteria (i.e., wide QRS complex, atrioventricular dissociation). In experiments in which the heart rate was lowered by carbachol, ventricular rhythms with cycle lengths longer than 150 ms (intrinsic cycle lengths of isolated mouse hearts) were considered ventricular escape beats and were not included in the analysis. The ventricular origin of abnormal beats on the ECG was verified on the optical map. Only ectopic beats with a visible full activation ring were used for the analysis.

### Computational modeling of DAD-like activity in the ventricles or PFs.

All modeling was conducted in openCARP (http://opencarp.org; free for noncommercial use) (40), a finite-element software custom designed to simulate electrophysiological phenomena at the cell, tissue, and organ scales. The Purkinje–myocardial junction was modeled as a geometric tissue expansion between 2 regions with distinct cell- and tissue-scale electrophysiological characteristics. Specifically, the model was a rectangular prism-shaped block of ventricular tissue (dimensions: 1 × 1 × 0.475 cm^3^) with a PF-like structure (1 cm × 500 μm × 125 μm), attached at a single junction (Supplemental Figure 5). Membrane kinetics were represented by a well-validated human ventricular action-potential model (41). To represent differences in excitability, the simulated sodium channel conductance was fixed at 0.5 times its default value in ventricular cells; the same parameter was fixed at 5 times in PF-like cells. Myofiber orientations in the PF region were uniform and parallel to the long axis of the structure; in ventricular tissue, myofibers ran in the perpendicular direction, as shown in Supplemental Figure 5. To account for heterogeneity of intercellular coupling between tissues, we adjusted conductivity values (PF vs. ventricular: longitudinal, 2.336 vs. 0.2336 S/m; transverse, 0.1761 vs. 0.01761 S/m).

To simulate DAD-like activity, the ventricular tissue was initialized in a refractory state, and then a short stimulus was applied to elicit a subthreshold response. The refractory steady state was obtained by pre-pacing the model at 2 Hz and then freezing-state variable values at the time point corresponding to the AP duration at 90% repolarization. We empirically calibrated a transmembrane current pulse (20 ms long; amplitude, 4.3740234375 pA/pF) that produced a transient depolarization without triggering a new AP. A buffer region of ventricular cardiomyocytes adjacent to the PF was modeled identically to other ventricular cells but without the electrical stimulus. To gauge how this affected the propensity for retrograde excitation, the buffer region’s radius was varied from 0 to 300 μm in 75 μm steps.

### Statistics.

Statistical analyses were performed using Prism, version 7.04 (GraphPad Software, Inc.). Statistical tests were used as reported in the figure legends. A *P* value of 0.05 was used as the threshold to reject the null hypothesis.

### Study approval.

The use of animals was approved by the Animal Care and Use Committee of Vanderbilt University (animal protocol no. M1600090-00 and M1600259-00) and performed in accordance with NIH guidelines.

## Author contributions

DJB, MF, RV, NGH, FJB, and MJW performed the experiments. DJB, MF, MJW, and BCK analyzed data. DJB, MF, and BCK wrote the manuscript. KP, GIF, and SG provided critical reagents and editorial input. CEG and PMB developed the computational model, conducted simulations, and analyzed related data. The order of the first authors was based on the extent of their scholarly contribution to the overall manuscript.

## Supplementary Material

Supplemental data

Supplemental video 1

## Figures and Tables

**Table 2:**
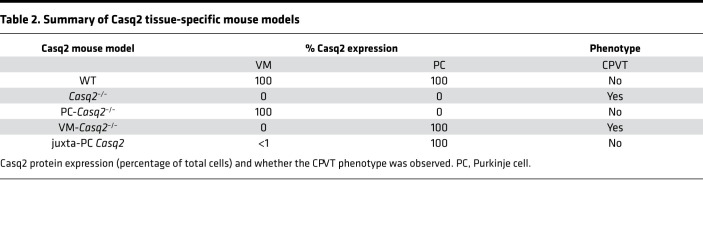
Summary of Casq2 tissue-specific mouse models

**Table 1:**
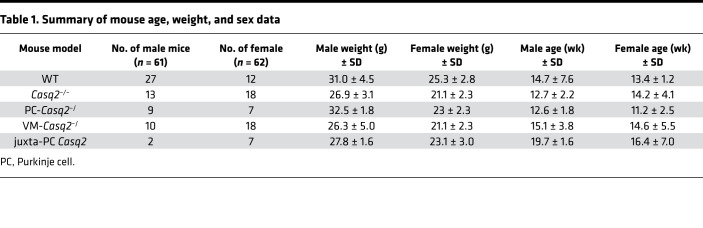
Summary of mouse age, weight, and sex data

## Version 1 01/06/2022

In-Press Preview

## Version 2 02/08/2022

Electronic publication
