# Supplementary material for: The Purkinje–myocardial junction is the anatomic origin of ventricular arrhythmia in CPVT
Source: JCI Insight. 2022 Feb 8;7(3):e151893. doi: 10.1172/jci.insight.151893 (PMC8855823; doi:10.1172/jci.insight.151893)
Supplement: Supplemental data [file jciinsight-7-151893-s201.pdf]

Data Supplement

The Purkinje-myocardial junction is the anatomical origin of ventricular arrhythmia in CPVT

Daniel J. Blackwell, Michela Faggioni, Matthew J. Wleklinski, Nieves Gomez-Hurtado, Raghav Venkataraman, Chelsea E. Gibbs, Franz J. Baudenbacher, Shiaoching Gong, Glenn I. Fishman, Patrick M. Boyle, Karl Pfeifer, Bjorn C. Knollmann

Supplemental figures 1-5
Supplemental table 1

Supplemental figure 1

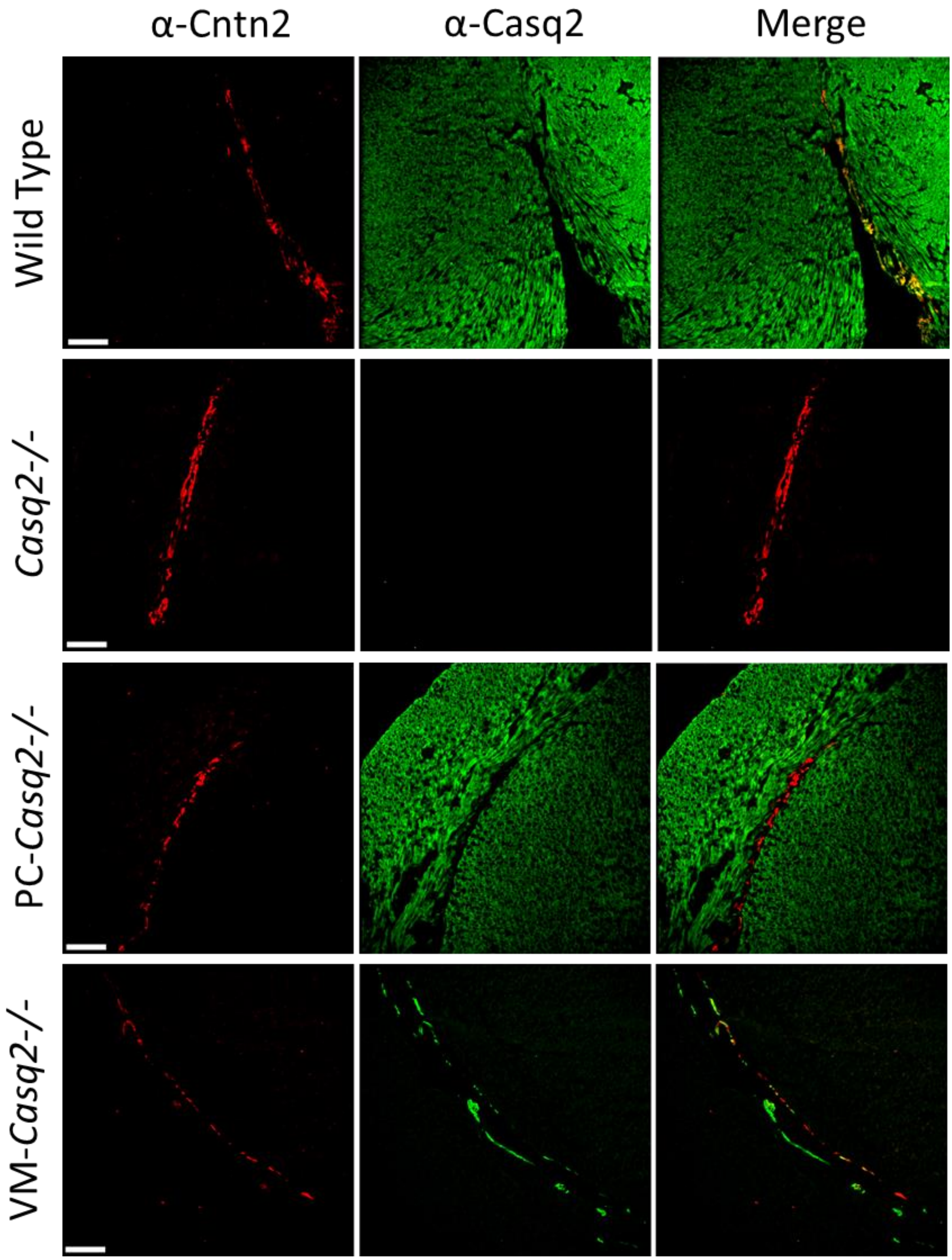

Supplemental Figure 1. Representative images of contactin-2 (Cntn2; a Purkinje cell maker) and calsequestrin-2 (Casq2) immunostaining in tissue-specific Casq2-/- mouse lines. Scale bar = 200 µm. PC = Purkinje cell; VM = Ventricular myocardium.

Supplemental figure 2

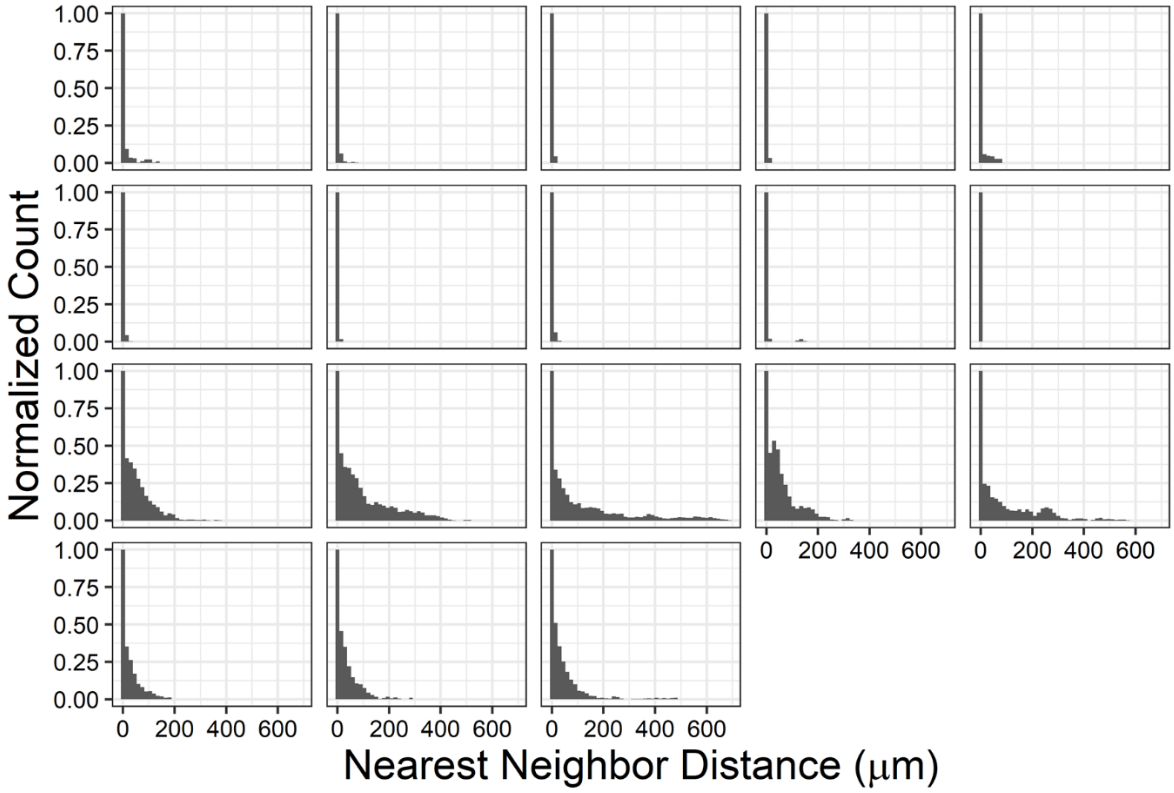

Supplemental Figure 2. Nearest neighbor distance (NND) distributions calculated on a pixel-by-pixel basis for cardiac calsequestrin (Casq2) staining relative to contactin-2 (Cntn2) staining for each heart shown in Figure 4. Data are displayed in 15 μm bins. Data were analyzed from a single 2-dimensional plane. n = 18 hearts.

Supplemental figure 3

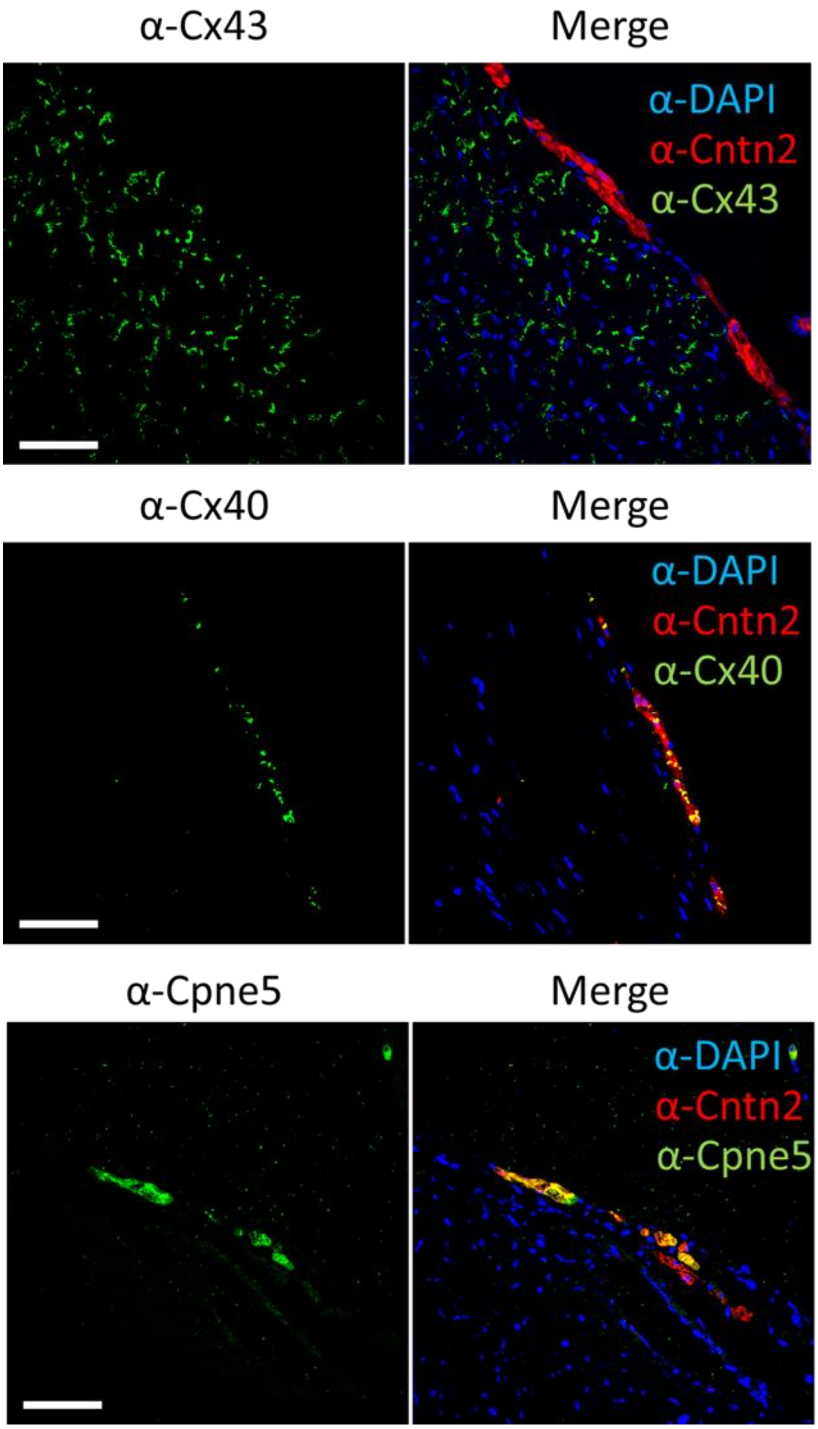

Supplemental Figure 3. Contactin-2 (Cntn2) positive cells are positive for the ventricular cardiomyocyte marker Connexin-43 (Cx43) and negative for the purkinje and transitional cell markers Connexin-40 (Cx40) and Copine 5 (Cpne5). Cx43 is a gap junction protein expressed only in the ventricular myocardium but not the atria and conduction system. Cx40 is another gap junction protein that is expressed in atrial myocytes as well as the His-Purkinje system including transitional cells. Cpne5, is a calcium-dependent membrane-binding protein that is expressed in the conduction system including transitional cells. Frozen sections from a C57BL/6 mouse heart tissue were stained. For each antibody, a representative 40x image of the antibody alone is shown (left panel) together with a merged image (right panel) of co-staining with DAPI (blue) and Cntn2 (red). Scale bar = 50 µm.

Supplemental figure 4

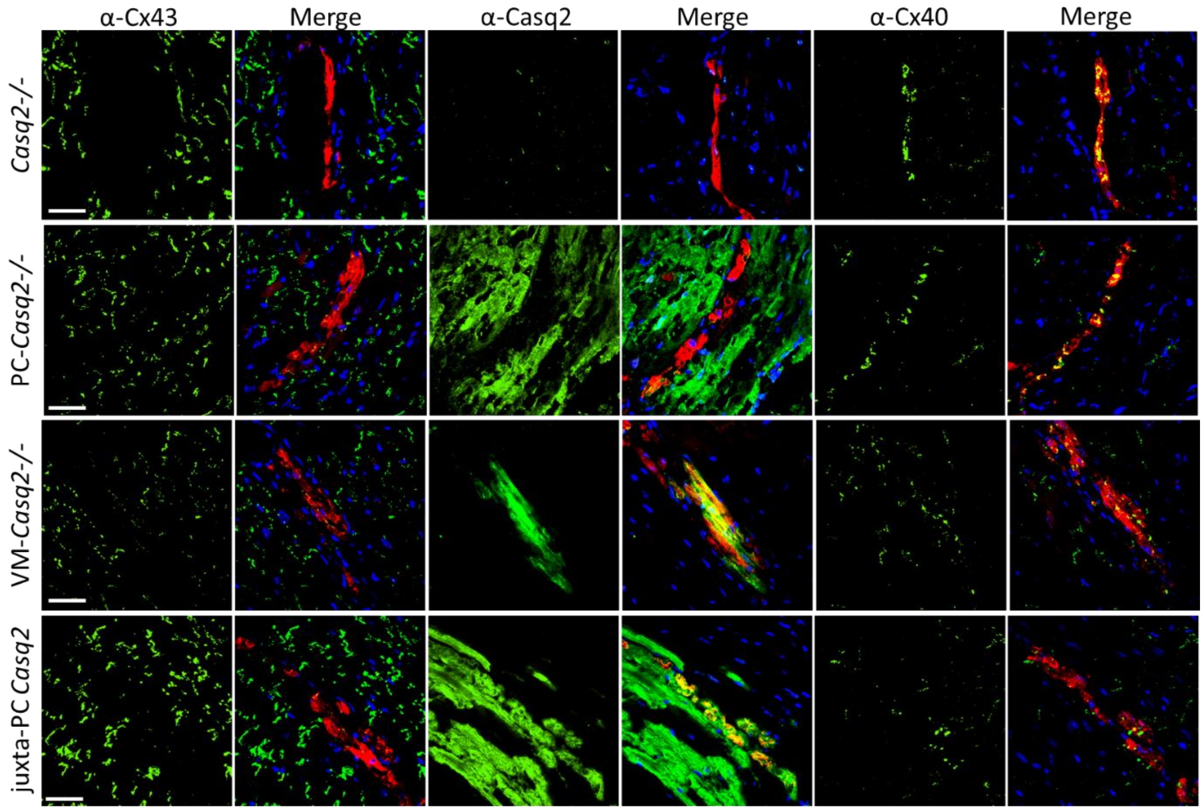

Supplemental Figure 4. Juxta-purkinje cells expressing calsequestrin-2 (Casq2) are positive for the ventricular cardiomyocyte marker Connexin-43 (Cx43) and negative for the purkinje and transitional cell markers Connexin-40 (Cx40) and Contactin-2 (Cntn2). Frozen sections from the hearts of each tissue-specific Casq2 mouse model were stained for the cardiac proteins mentioned above. For each antibody, a representative 40x image of the antibody alone is shown (left panel) together with a merged image (right panel) of co-staining with DAPI (blue) and Cntn2 (red). Scale bar = 50 µm.

Supplemental figure 5

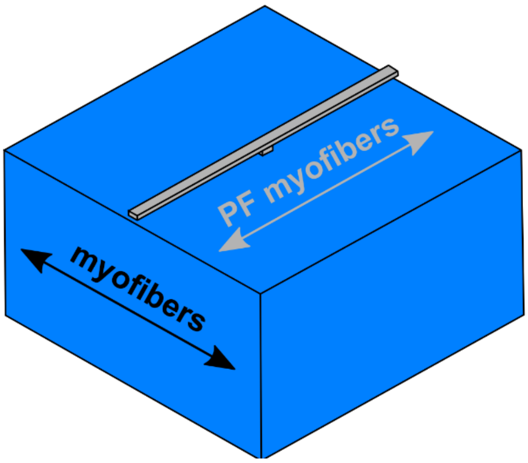

Supplemental Figure 5. Schematic representation of the computational model, with Purkinje fiber (PF) primed for delayed afterdepolarization (DAD)-like behavior and ventricular tissue ready to be excited (i.e., cell-scale ionic model conditions).

Supplemental table 1

Casq2 tissue model	Colocalization (%)		
	Cntn2>Casq2	Cx40>Cntn2	Cx43>Cntn2
Casq2 -/-	0	89	0
PC-*Casq2 -/-*	0	91	4
VM-*Casq2 -/-*	88	95	0
juxta-PC *Casq2*	87	89	1

Supplemental Table 1. Colocalization data of cardiac-specific proteins. Frozen sections from the hearts of each tissue-specific calsequestrin-2 (Casq2) mouse model were stained for Casq2, connexin-40 (Cx40), or connexin-43 (Cx43). All sections were co-stained with contactin-2 (Cntn2). Using the Just Another Co-localization Plugin (JACoP) in ImageJ, images were analyzed for the percent colocalization of various protein pairs. Within JACoP, images were assessed using the M1 and M2 coefficients. M1 is defined as the ratio of the "summed intensities of pixels from the image for which the intensity in the green channel is above zero" to the "total intensity in the red channel" and M2 is defined conversely for red. The output from the M1 and M2 coefficients ranges from 0 to 1 and is represented as a percent colocalization for the protein pair being analyzed.
